# Subtractive assembly for comparative metagenomics, and its application to type 2 diabetes metagenomes

**DOI:** 10.1186/s13059-015-0804-0

**Published:** 2015-11-02

**Authors:** Mingjie Wang, Thomas G. Doak, Yuzhen Ye

**Affiliations:** School of Informatics and Computing, Indiana University, Bloomington, IN 47405 USA; Department of Biology, Indiana University, Bloomington, IN 47405 USA; National Center for Genome Analysis Support, Indiana University, Bloomington, IN 47401 USA

**Keywords:** Comparative metagenomics, Subtractive assembly, Bloom filter, Type 2 diabetes

## Abstract

Comparative metagenomics remains challenging due to the size and complexity of metagenomic datasets. Here we introduce subtractive assembly, a de novo assembly approach for comparative metagenomics that directly assembles only the differential reads that distinguish between two groups of metagenomes. Using simulated datasets, we show it improves both the efficiency of the assembly and the assembly quality of the differential genomes and genes. Further, its application to type 2 diabetes (T2D) metagenomic datasets reveals clear signatures of the T2D gut microbiome, revealing new phylogenetic and functional features of the gut microbial communities associated with T2D.

**Electronic supplementary material:**

The online version of this article (doi:10.1186/s13059-015-0804-0) contains supplementary material, which is available to authorized users.

## Background

Metagenomics relies on the direct sequencing of an entire community of microbial organisms, but the results can be hard to disentangle [1]. Microbial communities vary in compositional complexity [2], from the simplest acid mine drainage microbial community with a few species to more complex microbial communities that may contain hundreds — even thousands — of microbial species (such as the human microbiome [3]). Even though many new methods and tools have been developed for analyzing metagenomic sequences, it remains a great challenge to infer the composition and functional properties of a microbial community from a metagenomic dataset, and to address causal questions, such as the impact of microbes on human health and diseases. Metagenomic assembly (the assembly of metagenomic samples) is one of the challenges. While assembly of a single genome using short reads has improved in recent years, even that remains an area of active improvement [4]. In the case of metagenomic datasets, it is difficult for conventional genome assemblers to deal with closely related strains and to distinguish true variations from sequencing errors [5]: using simulated Illumina reads from a 400-genome community, Mende et al. [6] found that relatively few of the reads were assembled, and of the contigs produced, 37 % were chimeric. Also, the varied depth of coverage across the individual chromosomes leads to ambiguity in assembly [7]. Finally, the sheer size of metagenomic datasets poses a challenge, as sufficient sequencing must be done to represent ever rarer members of the community [7]. But there is much to be learned by comparing metagenomic datasets sampled from different environments (or hosts): metagenomics can be used to reveal important connections between microbes and other aspects of life (such as human health and disease). A recent exemplar is the identification of a connection between microbes and type II diabetes [8]. Comparative metagenomics studies how environment and/or health correlate with microbial communities phylogenetically and functionally, using either 16S ribosomal RNA data or whole genome shotgun metagenomic sequence data [9]. Early studies compared the genomic diversity and metabolic capabilities across dramatically different metagenomes using barely assembled sequence data [10], while recent studies are more concerned with investigating how environmental or health features correlate with metagenomic differences using largely similar metagenomes [11–14].

Traditional comparative metagenomics begins with estimating biodiversity using short reads [15], or characterizing the biological and functional profiles based on known databases [16, 17]. Maillet et al. [18] proposed an approach that compares multiple metagenomic samples by efficiently identifying shared or similar reads based on *k*-mers and Jiang et al. [19] further developed several statistics that measure the dissimilarity between samples using sequence signatures (frequencies of *k*-mers) and applied them to metagenomics. It has also been reported that sequence signatures are similar for fragments from the same genome, but distinct between genomes [20]. Therefore, metagenomes with different microbial compositions tend to have distinctive sequence signatures, and the similarity and dissimilarity of metagenomes can be calculated using short reads without using any prior information.

Here we propose a subtractive assembly approach, a de novo method to compare metagenomes through metagenomic assemblies, aiming to achieve better assembly of the “differential” genomes for downstream analysis (e.g., to infer potential microbial markers associated with a human disease). For two or more metagenomes, reads that constitute the compositional difference are extracted from each metagenome based on sequence signatures. For example, we may define *k*-mers that occur ten times more frequently in one dataset than in the other as “signatures” that constitute the genomic difference; reads containing these signatures are likely to be from genomes that are more abundant or even unique in one of the two metagenomes. After read filtering, the complexity of the metagenome data sets can be greatly reduced, such that metagenome assembly using the extracted distinctive reads can be improved due to reduction in both biological diversity and data size. The compositional and functional difference of metagenomes can thus be characterized by the better-assembled contigs obtained from subtractive assembly.

For *k*-mer-based methods, a crucial step is the counting and storing of all *k*-mers. A number of efficient *k*-mer counting algorithms are publicly available [21–23]: BFCounter [22] adopts a bloom filter, making it quite memory efficient and thus most suitable for comparative metagenomics. We modified the C++ code of BFCounter to output reads with distinctive signatures. With simulated metagenomic datasets, we show that subtractive assembly can both effectively extract the reads from genomes that cause the compositional differences between metagenomes and improve metagenomic assembly for these genomes.

Our subtractive assembly is superficially similar to the method developed by Stranneheim et al. [24], which reduces the complexity of the metagenome assembly problem by filtering out reads that can be classified to known genomes, assuming that they are often of no interest. Our subtractive assembly approach takes advantage of the availability of metagenomic datasets of the same community under different conditions: when we are interested mostly in the differences between two (groups of) metagenomes, we can assemble only the differences by filtering out reads that are likely to have been sampled from species that are common to both samples. Our method is independent of reference genomes.

Type 2 diabetes (T2D) is one of the many diseases that have an associated microbial “profile”: it is associated with increased levels of streptococci, lactobacilli and *Streptococcus mutans* in oral samples [25]; *Lactobacillus* in gut microbiota is linked to obesity in humans, and weight gain for newborn ducks and chicks [26–28]; and Karlsson et al. [8] found that four *Lactobacillus* species and *S. mutans* are enriched in the gut microbiota of European women with T2D, using a large cohort of gut microbiome datasets. We applied our method to these gut metagenomes to see if our method could replicate the previous results, and perhaps further them: our subtractive assembly revealed new phylogenetic and functional features of the gut microbial communities associated with T2D.

## Results and discussion

We first tested subtractive assembly using simulated metagenomic datasets, and then applied it to the datasets from [8], to identify differential features of the T2D-associated microbiome. Our results show that the compositional difference of multiple metagenomic datasets could be detected using our *k*-mer-based method. Moreover, subtractive assembly utilizing only the reads that represent the compositional difference substantially reduced the complexity of the datasets and greatly improved the quality of the resulting assemblies, facilitating identifying compositional and functional differences between microbiomes. Application of our approach to the T2D datasets resulted in a large collection of genes that are uniquely found in the T2D-associated gut microbiomes, but which had not previously been identified.

### Evaluation of subtractive assembly: effectiveness of differential reads extraction and the requirement for abundance differences

**Fig. 1:**
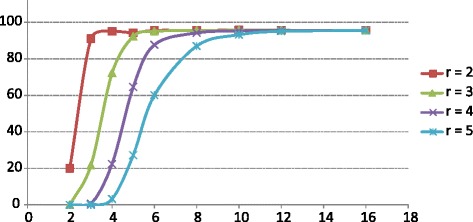
Fraction of the *S. thermophilus* LMD-9 genome assembled using subtractive assembly with different *k*-mer ratio parameters *r* (2–5; simulation 1). The x-axis shows the abundance ratio of this genome between samples and the y-axis shows the fraction (percentage) of the genome covered by contigs

We first tested the effectiveness of the *k*-mer-counting-based extraction of differential reads, using simulated metagenomic samples composed of five bacterial genomes (in three groups of five, four and three samples; Table 1; real microbiomes, such as the gut microbiomes which we analyze below, can be much more complex). In each group, S1 has a uniquely large proportion of *Streptococcus thermophilus* reads. For each of the groups, sample 1 (S1) was subtracted by each of the other samples (S2, S3 and so forth) and the remaining reads were used for assembly. The fold change of the *S. thermophilus* genome ranges from 2–16 (Table 1). We examined how the assembly coverage of the *S. thermophilus* reference genome changes when the parameters, including the actual abundance ratio of the genome in two metagenomes (or the fold change) and the *k*-mer ratio threshold used in the subtractive assembly, are changed (Fig. 1; for real metagenomes, we used an iterative subtractive assembly approach without fixing the *k*-mers ratio — see below). The results suggest that subtractive assembly can effectively detect the differential genome when the abundance ratio of the genome between two samples is about two times (or greater) the *k*-mer ratio threshold (parameter *r*) (on the other hand, when *r* decreases to < 2, significantly more reads from non-differential genomes are also extracted and subtractive assembly loses its power; Figure S1 in Additional file 1). For instance, 97.84 % (581,047 out of 593,858) of the reads from *S. thermophilus* LMD-9 were extracted and 95.03 % of the genome is covered by contigs when *r* = 2 and the simulated abundance of the *Streptococcus* genome is four times different in abundance between the two datasets. Based on this, we conclude that the *k*-mer ratio threshold needs to be set to *r = R/2* to effectively assemble a genome that is about *R* times more abundant in sample A than B, using subtractive assembly (i.e., A minus B). The simulation also suggests that the subtractive assembly approach can effectively capture genes with abundance changes of three-fold or more.

**Table 1:** Species composition of the artificial metagenomic samples in simulation 1

	Group 1					Group 2				Group 3		
	S1	S2	S3	S4	S5	S1	S2	S3	S4	S1	S2	S3
*Ferroplasma acidarmanus* fer1	1^a^	16	1	1	1	1	12	1	1	1	10	1
*Lactobacillus gasseri* ATCC 33323	2	2	16	2	2	2	2	12	2	2	2	10
*Pediococcus pentosaceus* ATCC 25745	4	4	4	16	4	4	4	4	12	4	4	4
*Prochlorococcus marinus* NATL2A	8	8	8	8	16	8	8	8	8	8	8	8
*Streptococcus thermophilus* LMD-9	16	1	2	4	8	12	1	2	4	10	1	2
RA ratio (*S*_*1*_ */S*_*i*_, *i* ! = 1)^b^		16^c^	8	4	2		12	6	3		10	5

^a^Relative abundance (*RA*) of the *F. acidarmanus* species in sample S1

^b^Relative abundance of the *S. thermophilus* genome in S1 relative to S2, S3 and so on. Pairs of datasets (S1 in each group, and another one in the same group) were subjected to subtractive assembly

^c^The relative abundance of the *S. thermophilus* genome in S1 versus S2

**Fig. 2:**
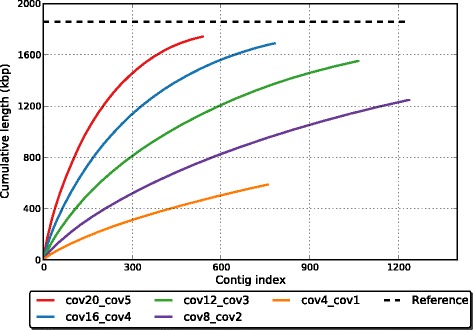
Comparison of the cumulative contig length of subtractive assembly at different sequencing depths of *S. thermophilus* LMD-9: 20× versus 5× (*red*), 16× versus 4× (*blue*), 12× versus 3× (*green*), 8× versus 2× (*purple*), 4× versus 1× (*orange*)

As shown above, our subtractive method can effectively recover reads originating from differential species between metagenomes. However, due to the random nature of shotgun sequencing, some regions of differential species may lack reads and are, therefore, poorly assembled, especially when the sequencing depth is not high. Here we tested subtractive assembly using simulated datasets with varying sequencing depth to demonstrate the impact of sequencing depth on the performance of subtractive assembly, using the same population structure as S1 or S4 from group 1 in simulation 1. We synthesized five pairs of datasets in which the sequencing depth ranges from 1–20× (Table 2): the sequencing depth for S1 ranges from 4–20× while it ranges from 1–5× for S4 (so in each pair of datasets, the relative abundance of *S. thermophilus* LMD-9 in S1 remains four times that in S4). Subtractive assembly (*r* = 2) was applied to each pair of datasets and we evaluated its performance according to the percentage of extracted reads and the fraction of the *S. thermophilus* genome assembled. As shown in Table 2, although the sequencing depth varies across the simulated datasets, the percentage of extracted reads was perfectly correlated with the expected ratio of differential reads (*R*^2^ = 0.9739), indicating that the performance of the subtraction step is mostly determined by the relative abundances of a genome between metagenomes. Not surprisingly, the quality of the final assembly is dependent on the sequencing depth (Fig. 2): when the sequencing coverage is low (e.g., 4×), only a small proportion of the differential genome can be assembled; but our method recovers nearly all of the differential positions when the sequencing depth is sufficiently high (e.g., 16×).

**Table 2:** Impact of sequencing depth on subtractive assembly for *S. thermophilus* LMD-9

Sequencing depth		Base coverage^b^ (%)	Extracted reads^c^ (%)	Assembled genome^d^ (%)
S1^a^	S4			
4×	1×	82.15	86.69	31.72
8×	2×	86.96	88.93	67.31
12×	3×	90.58	93.15	83.72
16×	4×	92.96	95.53	90.92
20×	5×	94.79	96.91	93.82

^a^The community structures of S1 and S4 are the same as in simulation 1 (group 1 in Table 1)

^b^Expected percentage of bases with ≥ 2 times sequencing coverage in S1 than in S4

^c^Percentage of reads extracted from the simulated *S. thermophilus* genome in S1

^d^Fraction of the genome assembled using the extracted reads by our subtractive assembly approach

### Evaluation of subtractive assembly: quality of the subtractive assembly when closely related species co-exist

**Fig. 3:**
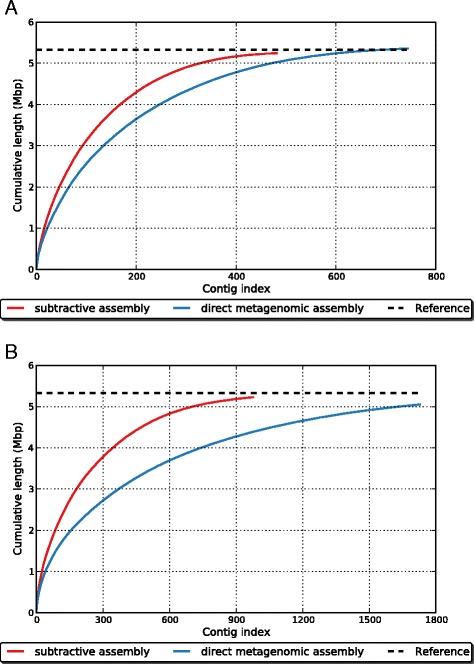
Comparison of the cumulative contig length between subtractive assembly (*red*) and direct metagenomic assembly (*blue*) of *R. palustris* HaA2 (simulation 2). The results assembled by IDBA-UD and MetaVelvet are shown in (**a**) and (**b**), respectively. On the x-axis, contigs are ordered from largest to smallest. The y-axis gives the size of the x largest contigs in the assembly

We then asked if subtractive assembly improves the assembly quality of metagenomes when closely related species exist in a sample, using another set of simulated metagenomic datasets consisting of five strains of *Rhodopseudomonas palustris* (Table 3). The dominant genome is *R. palustris* HaA2 in S1, while it is *R. palustris* CGA009 in S2. At the same time, the relative abundance of *R. palustris* HaA2 in S2 is substantially lower than that in S1: thus, *k*-mers representing the HaA2 genome will be identified and used for extracting reads from S1. For S1, subtractive assembly obtained longer contigs for the dominant *R. palustris* HaA2 genome than did direct assembly of the raw datasets, without much sacrifice of genome coverage (Fig. 3). Using contigs that are longer than 500 bp, the N50 is 21,374 in subtractive assembly, compared with 13,360 from the direct metagenomic assembly of metagenome 1; and the length of the largest contig is 113,404 bp compared with 95,495 bp. The genome coverage by contigs (total number of aligned bases in the reference divided by the genome size) is 98.3 % in subtractive assembly, compared with 98.6 % in direct assembly. The increased length of contigs comes with an acceptable number of misassemblies: the subtractive assembly produced three misassemblies (as reported by QUAST [29]), whereas the direct assembly produced one misassembly. The number of mismatches and indels, however, is decreased significantly in subtractive assembly of the distinctive reads: the number of mismatches is 394 with subtractive assembly and 2185 with direct assembly; and the number of indels is 8 with subtractive assembly and 80 with direct assembly.

**Table 3:** A pair of simulated metagenomic datasets containing five *R. palustris* strains (simulation 2)

Strain	Genome length	RA^a^		Sequencing depth	
		S1	S2	S1	S2
BisA53	5,505,494	3	3	18×	×
BisB18	5,513,844	3	3	18×	18×
BisB5	4,892,717	3	3	18×	18×
CGA009	5,459,213	0.1	5	0.6×	30×
HaA2	5,331,656	5	0.1	30×	0.6×

^a^Relative abundance. The two samples are S1 and S2

**Fig. 4:**
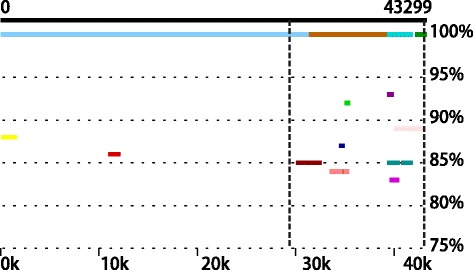
An example of the reduced fragmentation of contigs given by subtractive assembly. A long contig resulting from the subtractive assembly is broken into several shorter contigs when the subtraction step is not used (i.e., from the direct assembly). A number of contigs (highlighted by different colors) from the direct assembly are aligned to the long contig with different degrees of similarities. The polymorphic region is highlighted between two vertical dotted lines

A possible explanation for the superior performance of subtractive assembly in this simulation is that the subtraction step helps alleviate assembly problems caused by polymorphic regions (the regions that are similar, but not identical, in multiple genomes in the same metagenomic dataset). The sharing of homologous genes among different species is one of the known complicating factors that confuse de Bruijn graph-based assemblers (including IDBA-UD [30]) in metagenomic assembly, because they form tangled branches in the assembly graph. Since subtractive assembly targets the genomes that are more abundant (or unique) in one of the metagenomes, some of the closely related genomes will be filtered out during the subtraction step, reducing the complexity of the assembly problem. We compared the contigs from subtractive assembly and direct assembly using NUCMER [31] and confirmed the reduced fragmentation of contigs by the subtractive assembly resulting from the subtraction step. For instance, one 43,299-bp contig from subtractive assembly was fragmented into four contigs in direct assembly (Fig. 4). Positions around breakpoints recruited a number of contigs of different degrees of similarities, indicating that these are homologous regions shared by the different genomes in the metagenomic dataset (the five strains are only moderately similar to each other at a maximal unique matches index (MUMi) distance [32] of ~0.8 (0 ~ 1 scale), due to frequent genomic rearrangements).

We note that a comprehensive testing of all available assemblers is beyond the scope of this manuscript, but in addition to IDBA-UD [30] we tested MetaVelvet [33] and MEGAHIT [34]. To use MetaVelvet [33] for subtractive assembly on S1, we set the *k*-mer length as 51 for assembly (as suggested by the MetaVelvet manual). We saw an even greater improvement of assemblies by subtractive assembly, which is not surprising, since MetaVelvet originally generated shorter contigs for this dataset than IDBA-UD [30] (Fig. 3a, b), leaving more room for improvement. From the cumulative plot of contigs, we can see that contigs of the differential genome were longer if subtractive assembly is applied preceding metagenomic assembly. Using contigs that are longer than 500 bp, the N50 is 10,116 with subtractive assembly but only 4681 with direct metagenomic assembly, and the largest contig is increased from 76,007 bp with direct assembly to 98,570 bp with subtractive assembly. Even the genome coverage is improved with subtractive assembly, from 94.1 % to 97.6 %. MEGAHIT [34] is a more recently developed assembler, which also uses the iterative assembly strategy (similar to IDBA-UD [30]). Not surprisingly, its results were comparable to those from IDBA-UD (Figure S2 in Additional file 1), but more differences were observed between these two assemblers for the real T2D gut metagenomes, as shown below.

### Subtractive assembly of T2D gut metagenomes

We applied subtractive assembly to the analysis of T2D gut metagenomes, hoping to identify compositional/functional T2D-associated features of the human microbiome, as well as test our methods with datasets of naturally occurring complexity. We used 50 T2D datasets (a total of 129 gigabases) and all 43 normal glucose tolerant (NGT) datasets (90 gigabases). We did not include three T2D datasets that were outliers based on neighbor-joining clustering of the samples using a d_2_^S^ dissimilarity measure for *k* = 9 [19]. Table 4 shows the differential reads extracted for each group of samples (T2D or NGT). A large portion of the extracted reads represented unique *k*-mers, confirming the distinction between these two groups. For comparison, we also assembled the datasets directly (without the subtractive step). We tried two different approaches: assembling the metagenomic datasets individually, or co-assembling the pooled datasets. For clarity, we call the former direct assembly, and the latter direct co-assembly.

**Table 4:** A summary of the read extraction for the European women gut metagenomic datasets

k-mer ratio	NGT (gigabases)	T2D (gigabases)
2	14.48	12.66
4	2.48	1.66
6	0.55	0.42
8	0.17	0.13
10	0.04	0.05
(unique)	8.91	14.24

The subtractive assembly generated fewer contigs compared with the direct co-assembly — this is not surprising because the subtractive assembly focused on the differential portion. For direct co-assembly, as the pooled dataset is huge, we could only use MEGAHIT but not IDBA-UD (which used too much memory). However, we were able to co-assemble the distinctive reads (i.e., subtractive assembly) for the combined T2D and NGT metagenomes using either assembler because of the substantial data reduction in the subtraction step. Table 5 summarizes the assembly results for the T2D samples using the direct assembly and subtractive assembly approaches (the results show similar trends for the NGT datasets). In brief, subtractive assemblies are approximately one sixth (by IDBA-UD) to one half (by MEGAHIT) the length of direct assemblies, measured as the total length of contigs; and MEGAHIT produced more contigs, but its contigs are much shorter than IDBA-UD contigs (true for both direct assembly and subtractive assembly). There is no clear assembler winner in this case, but considering that IDBA-UD gave much longer contigs (and the memory usage is not a concern for our subtractive assembly approach due to the data reduction), we focus below on the downstream application of subtractive assembly results using IDBA-UD (but users can choose to use any of their favorite assemblers for subtractive assembly).

**Table 5:** A summary of the subtractive assembly and direct assembly results for T2D datasets

Metrics	Direct assembly		Subtractive assembly	
	IDBA-UD (individual)^a^	MEGAHIT (co-assembly)	IDBA-UD	MEGAHIT
Total contigs^b^	2,422,739	2,645,944	510,220	2,175,502
Total base	3,365,389,115	2,200,436,161	512,470,294	1,434,840,759
N50	2170	1054	1146	677

^a^The assemblies of individual samples were added for direct assembly by IDBA-UD

^b^Only contigs of at least 300 bp were considered for the statistics

### Subtractive assembly reveals compositional features of T2D gut metagenomes

**Fig. 5:**
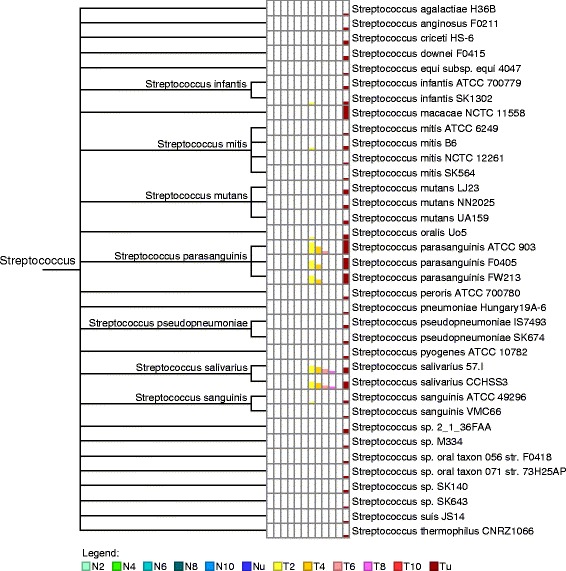
Compositional differences in *Streptococcus* species between the T2D group and NGT group. The genomes are identified in the T2D group while absent in the NGT group, through lowest common ancestor analysis. The first six columns and last six columns represent the iterative subtractive assemblies at *k*-mer ratio 2, 4, 6, 8, and 10, or that are unique for the NGT group and T2D group, respectively. The height of the *colored bars* in each column is proportional to the number of contigs that hit that taxon

To identify bacteria that are responsible for the difference between T2D and NGT gut metagenomes, we queried the contigs from subtractive assembly against the bacterial genomes (both complete and draft) deposited in National Center for Biotechnology Information (NCBI) using BLASTN [35]. MEGAN [16] was used to process the BLASTN [35] search results for taxonomic assignments of the contigs. About one half of the contigs were assigned to a reference genome in the database, and about one third of the unassigned contigs were identified by subtractive assembly but not by direct assembly. Consistent with previous studies [8], our results suggest enrichment of *Lactobacillus gasseri*, *Lactobacillus salivarius* and *S. mutans* in T2D datasets. However, we identified a greater variety of *Lactobacillus* and *Streptococcus* species (Fig. 5) as more abundant in the T2D group compared with the original analyses of these datasets [8]: for example, *Streptococcus parasanguinis* and *Streptococcus salivarius* are found to be enriched in the T2D datasets. We also identified genomes that are more abundant in the NGT group, including *Lysinibacillus fusiformis* ZC1, *Lysinibacillus sphaericus* C3-41, and *Pseudomonas putida* GB-1 (see Additional file 2: Figure S3 and Additional file 3: Figure S4 for all species that were uniquely detected in NGT and T2D, respectively). The roles of those genomes remain obscure and await further study.

Our results also show that many pathogenic bacteria (including *Actinomyces*, *Enterococcus faecalis* and *Rothia mucilaginosa*) are enriched in T2D datasets, which might be a consequence of the immunocompromised status of T2D patients. The association between enriched pathogens and diabetes has been consistently reported in previous studies: 42 % of published cases of perianal actinomycosis were from patients also diagnosed with diabetes [36]; diabetes mellitus was identified as a unique, independent risk factor for isolation of vancomycin-resistant *E. faecalis* [37] and made it easier for *R. mucilaginosa* to cause infections [38]; and another large-scale metagenomics study revealed higher levels of opportunistic pathogens in participants with T2D [39].

### Subtractive assembly delivers a large collection of unique or abundant genes in T2D gut metagenomes

Subtractive assembly provided us with genes that could not be (well) assembled by direct assembly of individual metagenomic samples, and we showed in simulation results that our method can improve metagenome assembly, so we further explored how this improvement would influence gene prediction and functional analysis results using the T2D datasets. Even though half of the contigs from subtractive assembly cannot be phylogenetically assigned, they could still be used for functional annotation, which may reduce the bias in the reference-based annotation. We compared subtractive assembly with direct assembly of individual samples (both assembled by IDBA-UD): out of 928,237 genes predicted from subtractive assembly, 141,104 genes (15 %) — among which there are 70,951 complete genes (including both a start codon and a stop codon) — cannot be found in the direct assemblies of T2D samples. Similarly, 149,321 (18 %) — among which 72,956 genes are complete — out of 821,130 genes are not included in the direct assemblies of NGT samples. Comparison of subtractive assembly results with the co-assembly results of the original datasets (both assembled by MEGAHIT) revealed improvement by subtractive assembly at comparable scales: 660,445 out of 2,978,267 genes (22 %) from subtractive assembly — among which there are 274,018 complete genes — cannot be found in the direct co-assemblies of T2D samples. Likewise, 350,997 out of 2,692,810 genes (13 %) — among which 132,557 are complete — are not included in the direct co-assemblies of NGT samples. These results suggest that co-assembly of the datasets (thanks to the development of memory-efficient assemblers such as MEGAHIT) helped to assemble more genes compared with assembly of individual datasets; but still data reduction by subtractive assembly helped to further improve the assembly results (no matter which assembler was used).

When we compare the genes we identified with the gene sets from the original analyses of the datasets [8], we see a significant number of new genes. The original analyses [8] resulted in a collection of 5,997,383 genes from all the samples including NGT samples and T2D samples (data retrieved upon request). Using 95 % sequence identity and 80 % coverage of the query as cutoffs, subtractive assembly resulted in 153,755 new genes (17 %) in the T2D group and 140,542 new genes (17 %) in the NGT group.

We are particularly interested in genes that are unique or more abundant in the T2D microbiomes. We annotated these genes according to the SEED classification system [38]. This gene set is enriched in subsystems including peptidoglycan biosynthesis, multidrug resistance efflux pumps, and lactose and galactose uptake and utilization (Table 6). Not surprisingly, the subsystems with the most hits are involved in energy harvesting (such as lactose and galactose uptake and utilization, and fructooligosaccharides and raffinose utilization), cell defense (e.g. peptidoglycan biosynthesis and multidrug resistance efflux pumps), and transport proteins (such as Ton and Tol transport systems and ECF class transporters), indicating a microbe-contributed elevated level of glycolysis/gluconeogenesis in the T2D group, consistent with previous observations that short chain fatty acids can lead to increased glycolysis/gluconeogenesis in the liver [40, 41]. We also identified sialic acid metabolism as enriched in the gut microbiome of T2D patients (Table 6); it has been reported that elevated sialic acid is strongly associated with T2D and raised serum sialic acid is a predictor of cardiovascular complications [42]. As the patients in this study are 70-year-old women, they may be in a relatively late stage of diabetes and therefore suffer from those complications.

**Table 6:** Top 20 SEED subsystems for genes identified uniquely by subtractive assembly in the T2D cohort

Rank	SEED subsystem	Number of genes
1	Peptidoglycan biosynthesis	635
2	Ton and Tol transport systems	451
3	Multidrug resistance efflux pumps	427
4	DNA replication	424
5	DNA repair, bacterial	364
6	Cell division subsystem	345
7	Lactose and galactose uptake and utilization	322
8	Restriction-modification system	322
9	Fructooligosaccharides and raffinose utilization	292
10	Glycerolipid and glycerophospholipid metabolism	276
11	Maltose and maltodextrin utilization	270
12	Sialic acid metabolism	257
13	Methionine degradation	253
14	Ribosome LSU bacterial	252
15	Glycolysis and gluconeogenesis	244
16	De novo pyrimidine synthesis	238
17	High affinity phosphate transporter	236
18	ECF class transporter	234
19	Purine conversion	222
20	Threonine and homoserine biosynthesis	220

We further narrowed down our selection of genes to those that are consistently more abundant across T2D microbiomes than in healthy controls (so can serve as dependable gene markers for T2D), considering that subtractive assembly can improve the assembly of those genes. To identify those consistently differential genes, we quantified the abundance of the genes using read-mapping (by BWA [43]), normalized by the total number of reads (per billion reads) in each sample, to identify the genes that are significantly enriched in the T2D group compared with the NGT group. Among the 141,104 differential genes that cannot be found in direct assemblies of T2D samples, 18,614 (13 %) were significantly enriched in all T2D samples, with q-value < 0.01 (Wilcoxon rank-sum test corrected by false discovery rate (FDR)). Although we observed similar rankings for the top subsystems, we saw increases in subsystems related to energy harvesting (e.g., the rank for the ‘fructooligosacchrides and raffinose utilization’ subsystem was increased from 7 to 2) using this more stringent collection of T2D differential genes that passed the multiple testing (Table 7). We list significantly T2D-enriched genes together with their annotations on our website (http://omics.informatics.indiana.edu/mg/SA/).

**Table 7:** Top 13 SEED subsystems for genes identified uniquely by subtractive assembly, and which passed a Wilcoxon rank-sum test with FDR correction, in the T2D cohort

Rank	SEED subsystem	Number of genes
1	Peptidoglycan biosynthesis	138
2	Fructooligosaccharides and raffinose utilization	103
3	Multidrug resistance efflux pumps	100
4	Cell division	89
5	Sialic acid metabolism	80
6	Gene cluster associated with Met-tRNA formyltransferase	77
7	Glycerolipid and glycerophospholipid metabolism	73
8	DNA repair, bacterial	73
9	Choline and bataine uptake and betaine biosynthesis	73
10	Murein hydrolases	72
11	Maltose and maltodextrin utilization	66
12	Beta-glucoside metabolism	62
13	Lactose and galactose uptake and utilization	62

### Example T2D signature subsystems and genes

**Fig. 6:**
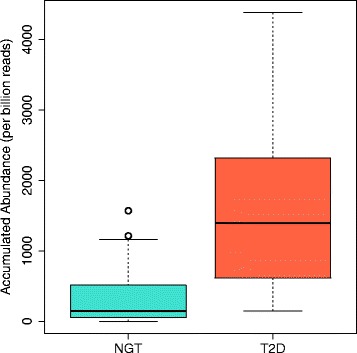
Abundance difference of the genes encoding beta-galactosidase between T2D and normal microbiomes (NGT). The abundance was measured as the number of reads that can be mapped to significantly T2D-enriched beta-galactosidase-encoding genes per billion reads. Note that we excluded 3 of 50 T2D samples with overly abundant beta-galactosidase genes (abundance > 6000) from the plot for clarity

Here we present a few involved subsystems and genes in detail. The first three subsystems involve utilization of fructooligosaccharides (FOS), maltose, lactose and galactose, and they are enriched in T2D women (ranked as 2, 11, and 13 in Table 7). For 11 out of 16 functional roles involved in the ‘Fructooligosaccarides and riffinose utilization’ subsystem, genes with differential abundances were identified (Table S1 in Additional file 1); detailed analysis of FIGfams in these three subsystems revealed an enrichment of several glycosidases with various substrate specificities (EC 3.2.1.-). For the utilization of FOS, there are at least three glycosidases with elevated levels in T2D: beta-glucosidase (EC 3.2.1.21), alpha-galactosidase (EC 3.2.1.22) and alpha-mannosidase (EC 3.2.1.24); for the utilization of lactose and galactose, beta-galactosidase (EC 3.2.1.23) is significantly increased in the T2D cohort (Fig. 6); similarly, alpha-glucosidase (EC 3.2.1.20) is increased, for enhanced utilization of maltose. We note that alpha-glucosidase inhibitors are well-established in the treatment of T2D, and work by reducing the absorption of carbohydrates from the small intestine [44]. Our work revealed other enriched glycosidases in T2D, which may provide alternative targets for the development of antidiabetic drugs.

**Fig. 7:**
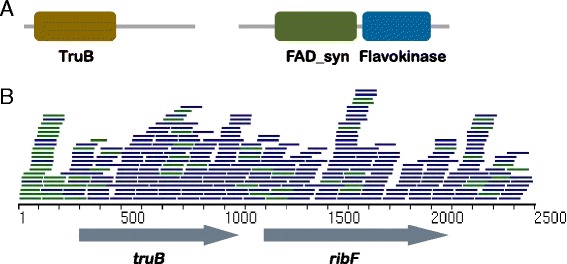
The *truB-ribF* operon identified by subtractive assembly as associated with T2D. **a** There are three domains in the operon: TruB encoded by *truB*; and flavokinase and FAD synthetase encoded by *ribF*. The flavokinase and FAD synthetase constitute the bifunctional prokaryotic riboflavin biosynthesis protein. **b** The genes *truB* and *ribF* in this operon are confirmed by read mapping. Reads mapped to the proper pair are colored in *blue* and mapped singletons are colored in *green*

The next two genes, *truB* (T2D_unique_8729_300_1012_+) and *ribF* (T2D_unique_8729_1193_2032_+), were found in the same contig assembled by subtractive assembly. The *truB* gene encodes the pseudouridylate synthase TruB (PF01509; 239 amino acids), and the *ribF* gene encodes a prokaryotic riboflavin biosynthesis protein (PF06574; 278 amino acids); the gene product of *ribF* has both flavokinase and adenine dinucleotide synthetase (FAD synthetase) activities (Fig. 7a). Flavokinases (EC 2.7.1.26) catalyze the conversion of riboflavin to FMN, while FAD synthetase (EC 2.7.7.2) adenylates FMN to FAD, together converting riboflavin to the catalytically active cofactors FMN and FAD [45]. By blasting the genes against the NR database [46], we identified the source genome to be *Blautia* sp. CAG:257 with 99 % identity and 98 % coverage of the query sequence. Karlsson et al. [8] also reported an abnormal level of riboflavin metabolism in the gut microbiome of T2D patients; however, they claimed that riboflavin metabolism was enriched in NGT women. We notice that their results may actually indicate the opposite (and so be consistent with our conclusion): they identified three KEGG (Kyoto Encyclopedia of Genes and Genomes) [47] protein families (KEGG Orthology groups) involved in riboflavin metabolism increased in NGT, while six other protein families were more abundant in T2D (shown in their supplementary table 12) [8]. The contig containing these genes was assembled from reads ‘unique’ to the T2D samples; read mapping confirmed that only a very few reads (59) from the NGT samples can be mapped to this 3450-bp contig (in contrast, 521 reads from T2D microbiomes can be mapped to this contig; Fig. 7b). This increase in FMN and FAD synthetase is consistent with the increased energy harvesting suggested above: FAD helps extract chemical energy by taking electrons from glucose during oxidative respiration.

The last gene (T2D_unique_70674_105_963_+) encodes a 285 amino acid protein with only one domain: MATE (PF01554; Multi antimicrobial extrusion protein). The protein belongs to one of the ten protein families (FIGfams) associated with the Multidrug resistance efflux pumps subsystem. This FIGfam (FIG 00000402) has the most hits for differential genes (342/427) among the ten FIGfams; members of this protein family extrude cationic drugs through an Na^+^-coupled antiport mechanism [48]. Taxonomic assignments of these proteins indicate a Firmicutes origin, especially *Clostridium*, *Lachnospiraceae* and *Erysipelotrichaceae*. It is known that mammalian MATE transporters mediate multidrug resistance by exporting diverse xenobiotic cations in the liver and kidney (MATE1 protein, for example, reduces the plasma concentrations of metformin, a widely prescribed oral glucose-lowering drug for the treatment of T2D, modulating its therapeutic efficacy [49, 50]), while bacterial MATE transporters act primarily as xenobiotic efflux pumps and have been reported to confer tigecycline resistance [48, 51, 52]. The elevated level of bacterial MATE pumps in the gut of T2D patients suggests a potential link between the disease and the gut microbiome through the elevated levels of medications, including antibiotics, taken by T2D patients [53, 54].

## Conclusions

Using both simulated and real metagenomes, we have shown that subtractive assembly improves the assembly of the differential genome between two metagenomes and facilitates downstream analysis. If the short reads from many genomes are directly assembled and annotated, it takes a tremendous amount of computational resources, as well as degrading the quality of the assembly. As a result, traditional comparative metagenomic approaches assemble each of the metagenomic samples independently, and then compare groups of samples by the common features shared among samples in each group. Instead, our method focuses on the compositional difference of the metagenome sets to be compared and therefore is well suited for large-scale comparative studies. Our method is able to consider a large number of samples simultaneously, which can also improve the assembly of differential genes, providing a complementary solution to existing comparative metagenomic approaches. We note that our subtractive assembly approach can effectively assemble genes with only an abundance difference of threefold or greater. However, genes with subtler abundance differences can still be discovered through the traditional comparative analyses of metagenomic datasets (using direct assembly approaches).

We developed our iterative subtractive assembly strategy to deal with situations where the compositional differences between metagenomes are unknown — which is typical. One advantage of this strategy is that it samples a spectrum of differences, aiding the assembly of genomes that are differential at various levels. However, if a user is interested in a certain degree of difference, a fixed *k*-mer ratio cutoff can be used in the subtractive assembly.

Our method currently compares two categories of metagenomes. It proves to be useful when we compare microbial communities between two treatment groups (such as healthy- versus T2D-hosted metagenomes). One future direction is to extend the method to allow comparison of multiple classes/treatments of metagenomes (e.g., sampled from multiple time points from the same environment; a control group and alternative treatments). A simple strategy is to apply subtractive assembly to all pairs of sample sets and then combine the results. We will also explore other approaches — for example, by correlating *k*-mers based on their frequency spectrum across samples for subtractive assembly — to make the best use of the multiple metagenomic datasets. Our approach to selecting consistently abundant genes related to T2D from differential genes assembled by subtractive assembly helps to narrow the gene list to the most promising ones (which are consistently differential between the two conditions according to the Wilcoxon rank-sum test with FDR correction).

Our analysis of T2D-hosted metagenomes indicates that subtractive assembly has a greater ability to detect differences than did previous analysis of the same data sets. But in general, we confirm that T2D-associated metagenomes have an increased ability to harvest energy from diverse carbohydrates, as other studies have shown. The enrichment of various glycosidases in T2D microbiomes suggests alternative targets for the development of antidiabetic drugs (alpha-glucosidase inhibitors are well-established in the treatment of T2D). The prevalence of *Blautia* sp. metabolism and Firmicutes-associated MATE xenobiotic efflux pumps seem to be exciting leads deserving of further study. We believe that our subtractive assembly approach can be applied to other datasets (e.g., the more recent liver cirrhosis datasets [55]) to reveal the association between microbial communities and other human diseases.

## Materials and methods

### Counting *k*-mers with the aid of a bloom filter

The bloom filter is a probabilistic data structure for determining whether an element belongs to a sparse set [23, 24, 56], using a number of hash functions to map the elements to the fixed bit space of the filter. Thus, false positives can occur when the bits for an element are shared by other elements. In other words, the bloom filter is a trade-off between memory usage and allowable false positives: suppose *n k*-mers are stored in a bitmap of size *m* using *d* hash functions, then the optimal value of *d* that minimizes the false positive rate is (*m/n)*ln(2) [22, 57]. As a fixed number of bits are used for each element, the complexity of inserting or querying an element is constantly O(d).

Bloom filters are memory efficient; however, the actual memory usage depends on the hash tables used for recording the number of occurrences of each *k*-mer. We modified the implementation of BFCounter (version 0.2) [22], following their principle of ruling out singletons of all *k*-mers encountered. A bloom filter *B* and a simple hash table *T* are adopted to store and count *k*-mers. The bloom filter *B* is used to store all existing *k*-mers, of which only *k*-mers observed twice or more are inserted into the hash table *T*. With the information stored in hash table *T*, we are able to calculate the distinctive sequence signatures for each metagenome. To detect the compositional differences of compared metagenomes, a *k*-mer ratio parameter *r* is employed to filter for *k*-mers that are more abundant or unique in one of the metagenomes. For example, if we set *r* = 10, we will only keep *k*-mers that occur at least ten times more frequently in metagenome A compared with metagenome B as differential *k*-mers representing metagenome A; the genomic differences between the two metagenomes are likely to be built using those signatures. We note that *k*-mer counts are normalized by the total bases in the corresponding metagenomic dataset, so that the *k*-mer ratio is not biased toward the larger metagenomic dataset.

### Read extraction based on sequence signatures and subtractive assembly

Reads made up of differential *k*-mers are from genomes that are most associated with the environmental conditions of interest (assuming a lack of confounding differences). Maillet et al. [18] considered two sequences *similar* if and only if they share at least a number of non-overlapping *k*-mers. Different from their approach, here we define reads containing at least a certain percentage (default 50 %) of differential *k*-mers as the *distinctive* reads. The reads satisfying this requirement are extracted and employed for metagenomic assembly.

IDBA-UD (version 1.0.9) [30] was adopted as the metagenomic assembler, following read extraction in subtractive assembly. It has been demonstrated that IDBA-UD achieves longer contigs with higher accuracy by taking into consideration the uneven sequencing depth of metagenomic sequencing technologies [29, 30]. We adopted the default options for IDBA-UD’s parameter settings: a minimum *k*-mer size of 20 and maximum *k*-mer size of 100, with 20 increments in each iteration. For comparison purposes, we also used IDBA-UD (using the same set of parameters) to assemble individual metagenomes without applying the subtraction step (referred to as the *direct* assembly approach). In addition, we tested MetaVelvet (version 1.1.01) [33] and MEGAHIT (version 0.2.1) [34]. In principle, however, any metagenomic assembler can be used for subtractive assembly.

### Iterative subtractive assembly

When subtractive assembly is applied to real metagenomic samples, we may choose a small *k*-mer ratio cutoff (e.g., 2), due to the unknown degree of compositional difference between the groups of samples being compared. Alternatively, we can iteratively extract reads using a series of *k*-mer ratio cutoffs. For the gut metagenomic datasets used in our study, the maximum ratio was set to 10 and the minimum 2, with a step value of 2. Besides this, we separately extracted reads characterized by unique *k-*mers (*k*-mers that occur in only one of the groups of samples): unique *k*-mers were first identified in each group and the corresponding distinctive reads were extracted; then non-unique *k*-mers that were more frequent in one group than the other were identified and the distinctive reads were extracted, starting from a *k-mer* ratio of 10, then 8, and so on. The stratification by iterative assembly provides more information on the compositional difference between two metagenomes, without any prior knowledge.

### Annotation of contigs from subtractive assembly

Contigs that are at least 300 nucleotides long were phylogenetically annotated by query against the bacterial genomes (both complete and draft genomes) deposited in the NCBI through BLAST searches [35]. BLAST results were then used for the assignment of lowest common ancestor by MEGAN (version 4) [16], with a minimum bit score (Min Score) of 80 and minimum contig support (Min Support) of 5.

Protein coding genes were predicted from the contigs using FragGeneScan [58]. We are interested in the protein coding genes covered only by subtractive assembly, and consider that a gene belongs to this category if there is no equivalent gene that covers at least 20 % of the gene with 90 % or higher sequence identity (based on RAPSearch2 [59]) in the direct assemblies of any individual metagenome. These genes were assigned to functional categories, including SEED subsystems [60]. We used myRAST (version 36; downloaded from http://blog.theseed.org/downloads/myRAST-Intel.dmg) for the SEED subsystem annotation.

To further validate the differential genes, we mapped the original short reads of each sample onto the genes that are enriched in the T2D cohort and normalized the coverage by the total number of reads in each sample. Based on the coverage of each differential gene in each sample, the significance of each candidate differential gene was tested by computing a one-sided *p* value using the R ‘wilcox.test’ function and correcting for multiple testing using false discovery rate (*q*-value) computed by the tail area-based method of the R ‘fdrtool’ package [61]. The fdrtool has been used for similar purposes in metagenomics projects [62–64].

### Simulated metagenomes

We carried out two simulations to test if our subtractive assembly approach can efficiently detect compositional differences between metagenomes (and the minimum abundance ratio for the difference to be detected), and improve assembly quality (especially when closely related species co-exist in a community).

In simulation 1, we simulated three groups of metagenomic datasets using five bacterial genomes from the FAMeS dataset [65]: *Ferroplasma acidarmanus* fer1, *Lactobacillus gasseri* ATCC 33323, *Pediococcus pentosaceus* ATCC 25745, *Prochlorococcus marinus* NATL2A, and *S. thermophilus* LMD-9. MetaSim (version 0.9.1) [66] was used to simulate reads from the genomes. In each group, the first sample (S1) was compared with each of the remaining samples in the same group for subtractive assembly. The relative abundances of the five genomes in each sample are shown in Table 1. In these samples, we only changed the abundances of the *S. thermophilus* genome and another genome, to keep the ratio of relative abundance for the *S. thermophilus* genome in the range of 2–16. This enables us to evaluate whether our method can effectively detect the compositional difference between metagenomes by focusing on a single genome (*S. thermophilus*). We applied the iterative subtractive assembly strategy to analyze this set of simulated datasets (*k*-mer ratio parameter *r* was set to be 2, 3, 4, or 5). After the subtractive assembly, we calculated the fraction of the *S. thermophilus* genome covered by contigs using QUAST [29] and MUMer [31]. In all the samples, the sequencing depth of the *Streptococcus* genome was designed to be between 30× and 40 × .

In simulation 2, we simulated a pair of metagenomic samples (S1 and S2) using five different *R. palustris* strains (Table 2). The *R. palustris* HaA2 genome is dominant in sample 1 (S1) and is the focus of this simulation. We set the *k*-mer ratio parameter to *r* = 2 for the subtractive assembly (S1 minus S2). Sample 1 was also used for direct metagenomic assembly using IDBA-UD [30]. Assemblies from both subtractive assembly and direct assembly were compared with the reference genomes using QUAST [29]. We used various metrics for assembly evaluation, including the cumulative length of contigs, N50, and size of the largest contig.

### Real metagenomes

We chose the large collection of gut metagenomic datasets derived from two groups of 70-year-old European women, one group of 53 with T2D and the other a matched group of healthy controls (NGT group; 43 participants) [8]. This collection of metagenomes is ideal for testing our subtractive assembly approach: only two groups were involved (T2D versus healthy) and each group contains many large metagenomic datasets. We pooled the T2D samples and NGT samples separately for subtractive assembly.

### Availability

Our tools for subtractive assembly are available for download at sourceforge (https://sourceforge.net/projects/subtractive-assembly/). We also make available the subtractive assembly results of the T2D metagenomes, including the set of genes that are uniquely or more abundantly found in T2D genomes, along with their annotations at http://omics.informatics.indiana.edu/mg/SA/.

## Additional files

Additional file 1: Table S1. Functional roles involved in the ‘Fructooligosaccharides (FOS) and Raffinose Utilization’ subsystem. **Figure S1.** Percentage of extracted reads from non-differential genomes by subtractive assembly on S1 vs. S2 in Group 2 of Simulation 1 (see Table 1 for more information). The x-axis shows the values of *k*-mer ratio parameter *r* (2 to 5) and y-axis shows the fraction (%) of reads from non-differential genomes in the extracted reads. **Figure S2** Comparison of the cumulative contig length between subtractive assembly (red) and direct assembly (blue) of *R. palustris* HaA2 (Simulation 2) by using MEGAHIT as the assembler. On the x-axis, contigs are ordered from largest to smallest. The y-axis gives the size of the x largest contigs in the assembly. (PDF 124 kb) Additional file 2: Figure S3. Species uniquely detected in the NGT group. (PDF 116 kb) Additional file 3: Figure S4. Species uniquely detected in the T2D group. (PDF 141 kb)

## Abbreviations

bp: base pair
FDR: false discovery rate
FOS: fructooligosaccharide
NCBI: National Center for Biotechnology Information
NGT: normal glucose tolerance
T2D: type 2 diabetes
