# Supplementary material for: Subtractive assembly for comparative metagenomics, and its application to type 2 diabetes metagenomes
Source: Genome Biol. 2015 Nov 2;16:243. doi: 10.1186/s13059-015-0804-0 (PMC4630832; doi:10.1186/s13059-015-0804-0)
Supplement: Additional file 1: Table S1. — Functional roles involved in the ‘Fructooligosaccharides (FOS) and Raffinose Utilization’ subsystem. Figure S1. Percentage of extracted reads from non-differential genomes by subtractive assembly on S1 vs. S2 in Group 2 of Simulation 1 (see Table 1 for more information). The x-axis shows the values of k-mer ratio parameter r (2 to 5) and y-axis shows the fraction (%) of reads from non-differential genomes in the extracted reads. Figure S2 Comparison of the cumulative contig length between subtractive assembly (red) and direct assembly (blue) of R. palustris HaA2 (Simulation 2) by using MEGAHIT as the assembler. On the x-axis, contigs are ordered from largest to smallest. The y-axis gives the size of the x largest contigs in the assembly. (PDF 124 kb) [file 13059_2015_804_MOESM1_ESM.pdf]

This document contains Supplementary Table 1, Figure 1 and Figure 2.

Subtractive assembly for comparative metagenomics, and its application to type 2 diabetes metagenomes

Mingjie Wang, Thomas G. Doak, Yuzhen Ye

Supplementary Table 1. Functional roles involved in the 'Fructooligosaccharides (FOS) and Raffinose Utilization' subsystem.

Column	Abbrev	Functional Role	#Hits
1	MsmR	MSM (multiple sugar metabolism) operon regulatory protein	3
2	MsmE	Multiple sugar ABC transporter, substrate-binding protein	8
3	MsmF	Multiple sugar ABC transporter, membrane-spanning permease protein MsmF	4
4	MsmG	Multiple sugar ABC transporter, membrane-spanning permease protein MsmG	4
5	MsmK	Multiple sugar ABC transporter, ATP-binding protein	6
6	SacA	Sucrose-6-phosphate hydrolase (EC 3.2.1.B3)	10
7	GtfA	Sucrose phosphorylase (EC 2.4.1.7)	8
8	DexB	Glucan 1,6-alpha-glucosidase (EC 3.2.1.70)	0
9	RafR	Raffinose operon transcriptional regulatory protein RafR	0
10	RafB	Raffinose permease	0
11	LacY	Lactose permease	0
12	Aga	Alpha-galactosidase (EC 3.2.1.22)	20
13	yesM	Two-component sensor kinase YesM (EC 2.7.3.-)	5
14	Man	Alpha-mannosidase (EC 3.2.1.24)	5
15	BG	Beta-glucosidase (EC 3.2.1.21)	30
16	Hyl	Hyaluronoglucosaminidase (EC 3.2.1.35)	0

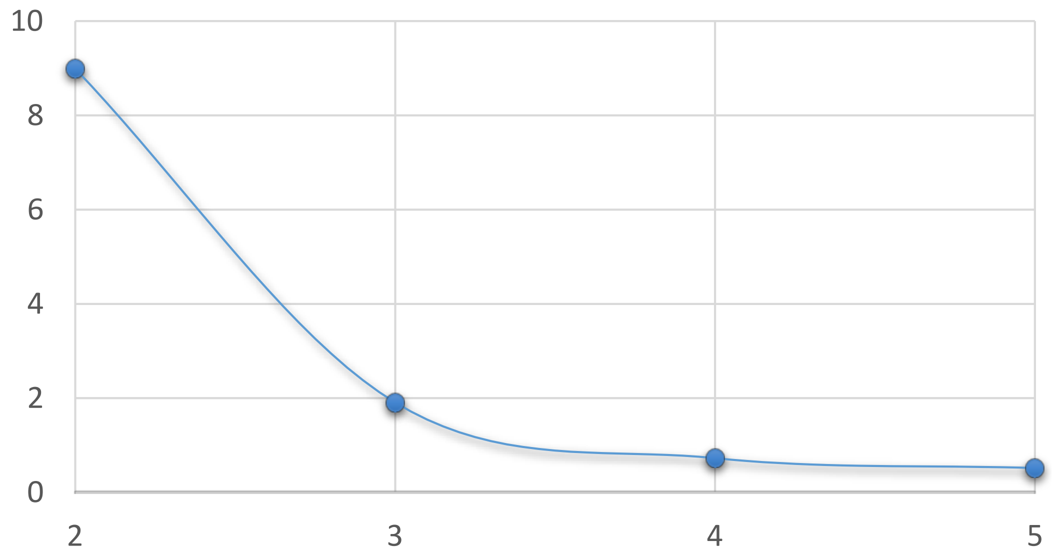

Supplementary Figure 1. Percentage of extracted reads from non-differential genomes by subtractive assembly on S1 vs. S2 in Group 2 of Simulation 1 (see Table 1 for more information). The x-axis shows the values of *k*-mer ratio parameter *r* (2 to 5) and y-axis shows the fraction (%) of reads from non-differential genomes in the extracted reads.

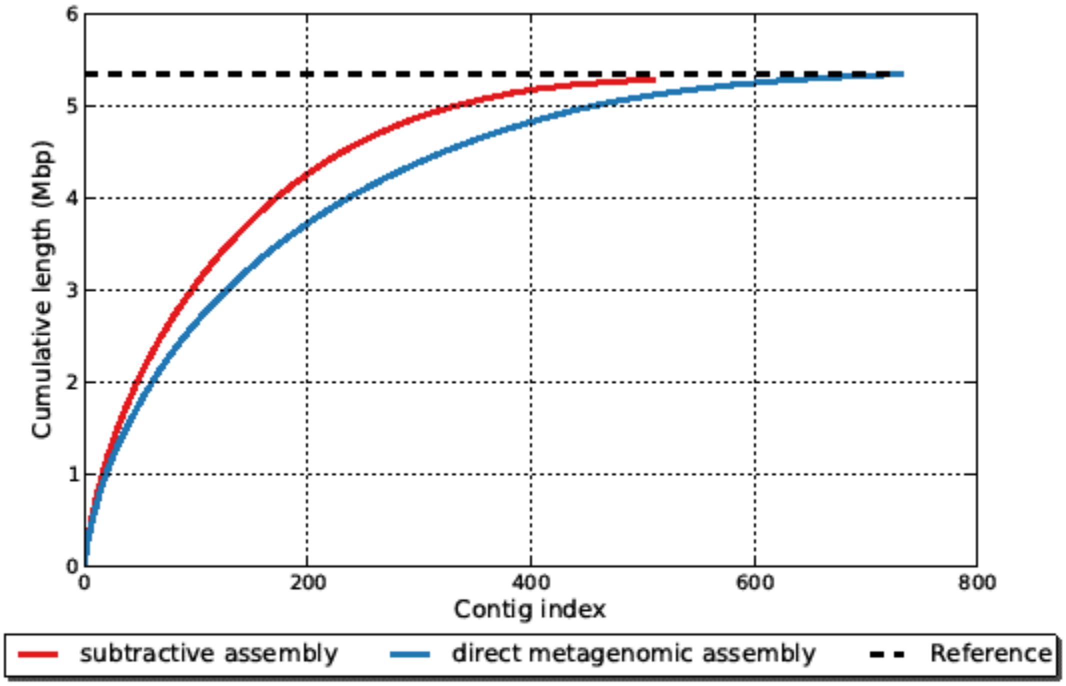

Supplementary Figure 2.Comparison of the cumulative contig length between subtractive assembly (red) and direct assembly (blue) of *R. palustris* HaA2 (Simulation 2) by using MEGAHIT as the assembler. On the x-axis, contigs are ordered from largest to smallest. The y-axis gives the size of the x largest contigs in the assembly.
