# Supplementary figures and images for: Subtractive assembly for comparative metagenomics, and its application to type 2 diabetes metagenomes
Source: Genome Biol. 2015 Nov 2;16:243. doi: 10.1186/s13059-015-0804-0 (PMC4630832; doi:10.1186/s13059-015-0804-0)

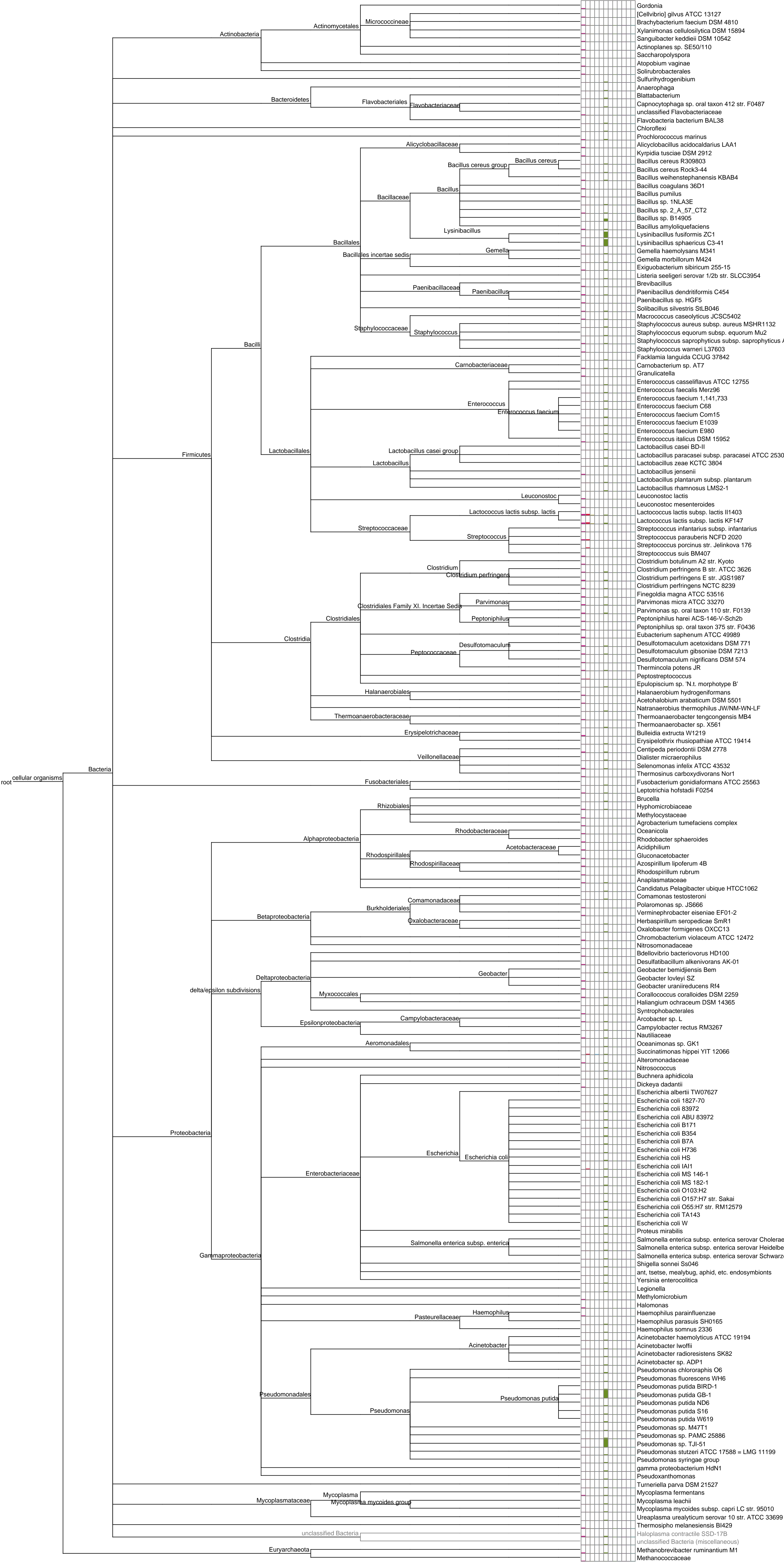

Legend:

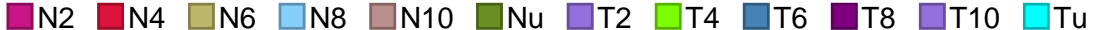

Supplement: Additional file 2: Figure S3. — Species uniquely detected in the NGT group. (PDF 116 kb) [file 13059_2015_804_MOESM2_ESM.pdf]

cellular organisms  
root

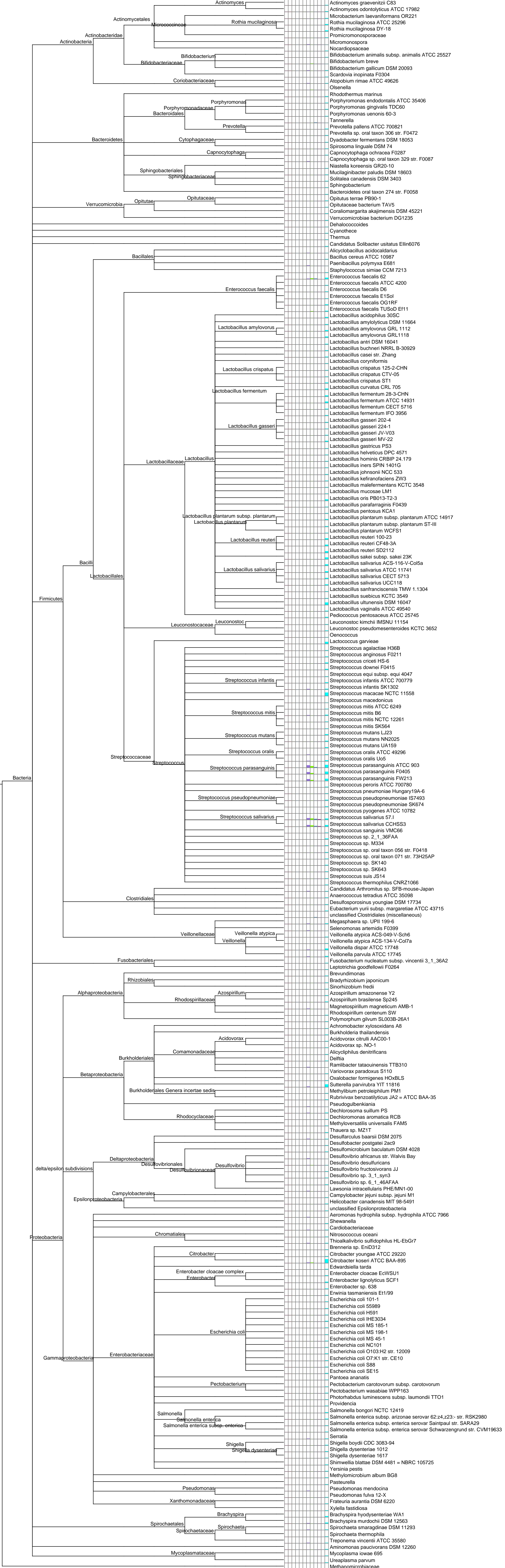

Legend:

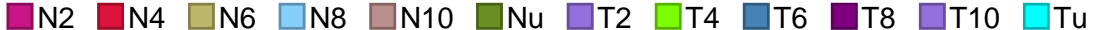

Supplement: Additional file 3: Figure S4. — Species uniquely detected in the T2D group. (PDF 141 kb) [file 13059_2015_804_MOESM3_ESM.pdf]
